# Magnitude and associated factors of caesarean section deliveries among women who gave birth in Southwest Ethiopia: institutional-based cross-sectional study

**DOI:** 10.1186/s13690-021-00682-5

**Published:** 2021-09-02

**Authors:** Ayenew Mose, Haimanot Abebe

**Affiliations:** 1 Department of Midwifery, College of Medicine and Health Science, Wolkite University, P.O.Box; 07, Wolkite, Ethiopia; 2 Department of Public Health, College of Medicine and Health Science, Wolkite University, Wolkite, Ethiopia

**Keywords:** Caesarean section, Magnitude, Associated Factors, Southwest Ethiopia

## Abstract

**Background:**

Caesarean section is a life-saving comprehensive obstetric procedure of women and newborn performed during childbirth-related complications and should be universally accessible for all pregnant mothers globally. Appropriate use of caesarean section can reduce maternal and perinatal mortality. However, inappropriate use of caesarean section can negatively affect infant health, women health, and future pregnancies. The magnitude and factors associated with caesarean section delivery were not consistent and will vary between different hospitals of Ethiopia. Hence, this study aimed at assessing the magnitude and factors associated with caesarean section deliveries in Southwest Ethiopia.

**Methods and Materials:**

An institutional-based cross-sectional study was conducted from January 1 to February 29, 2020. A systematic random sampling technique was used to select 551 study participants. A pretested, structured, and face-to-face interview was used to collect data. Data were entered into Epi-data version 4.2.0 and exported to SPSS version 23 for analysis. Bivariate and multivariate analyses were used to identify factors associated with caesarean section deliveries. *P* values < 0.05 result were considered as a statistically significant association.

**Results:**

The magnitude of caesarean section deliveries was found to be 32.5 % (95 % CI; 28.6%-36.7 %). Mothers resided in an urban area [AOR = 2.58, (95% CI; 1.66–4.01)], multiple pregnancies [AOR = 3.15, (95% CI; 1.89–5.23), malpresentation [AOR = 3.05, (95% CI; 1.77–5.24)], and previous history of caesarean section [AOR = 3.55, (95% CI; 2.23–5.64) were factors associated with caesarean section deliveries.

**Conclusions:**

Caesarean section deliveries were found high in the study area. Mothers resided in an urban area, multiple pregnancies, malpresentation, and previous history of caesarean section were factors associated with caesarean section deliveries. Therefore, counselling of mothers on the risk of giving birth through elective caesarean section without absolute and relative medical indications and giving enough time for the trial of vaginal birth after caesarean section are recommended.

## Background

Caesarean section (CS) is a comprehensive obstetric life-saving procedure of women and newborn performed during pregnancy and childbirth-related complications and should be universally accessible for all pregnant mothers globally [1]. Appropriate and timely use of CS can reduce maternal and perinatal morbidity and mortality. However, inappropriate use of caesarean section increased the risk of short-term and long-term maternal complications that can negatively affect infant health, women health, and future pregnancies [1, 2]. Currently, caesarean section is rising at an alarming rate, often for non-medical indications, has become a major public health concern globally [3].

A systemic review and meta-analysis conducted by Sobhy S, et al., showed that the risk of women dying after caesarean sections in developing countries was a hundred times higher than in developed countries. The burden was higher in Sub-Saharan Africa indicated that 1 out of 100 women died after CS, 82.5 stillbirths, and 100.4 perinatal deaths per 1000 caesarean sections have occurred [4]. Unnecessary use of CS without medical indication and lack of quality obstetric health care service after surgery were the major reasons for women death, stillbirth, and perinatal death [4, 5]. In 2015, the World Health Organization (WHO), suggested CS should be performed only when it is medically necessary, and more than 10-15 % of CS rates have not shown any potential benefits in the reduction of maternal and neonatal mortality [6].

The magnitude of caesarean section was varied across different countries. For instance, studies done in Latin American and Caribbean regions 40.5 % [2], United states 32 % [7], South Africa 42.4 % [8], South India 32.6 % [9], Tanzania 27 % [10], Sri Lanka 25.13 % [11], United Arab Emirates 30.2 % [12], Bangladesh 13 % [13], and in Ethiopia between 20.2 and 38.3 % of mothers were undergone caesarean section [14–20].

The most commonly cited factors of performing caesarean section were antepartum haemorrhage, fetal macrosomia, previous history of caesarean section, urban residence, and abnormal presentation were reported [13, 15–19]. Failed induction of labour, non-reassuring fetal heart rate pattern, and failure of labour progress were reported indications of performing caesarean section [8, 15, 18, 21].

The factors associated with caesarean section are multifactorial, complex, and will vary between different hospitals of Ethiopia. Besides, most of the above-published studies were conducted in a single hospital, used secondary data might affect by incomplete /missing files. Additionally, they were lack assessing the leading indications of caesarean section. Moreover, there was limited information regarding the magnitude and factors associated with caesarean section in the study area. Hence, this study aimed at assessing the magnitude, leading indications, and factors associated with caesarean section deliveries in Southwest Ethiopia. This study will have paramount importance for hospitals, zonal health offices, and policymakers to upgrade quality maternal and neonatal obstetric health care services provision.

## Methods and materials

### Study area, design and period

An institutional-based cross-sectional study was conducted from January 1 to February 29 in Gurage zone hospitals, Southwest Ethiopia. According to the 2011 census conducted by the Central Statistical Agency of Ethiopia (CSA) report, the zone has a total population of 8,556,964, of whom 4,278,482 are men and 3,422,785.6 women. In the near future, the total population of Gurage zone was projected to 6.5 million [20]. Gurage zone is located 187 km southwest of Addis Ababa the capital city of Ethiopia. Gurage zone has a total of 5 hospitals, 72 health centres, and 402 health posts that provides maternal and neonatal health care services for the catchment population. All 5 hospitals were providing comprehensive obstetric health care service and included in this study. Antenatal care, delivery, and postnatal care service were provided free of charge in the study area.

### Population and sampling

All mothers who gave birth in Gurage zone hospitals were considered as the source population and mothers who were selected using systematic random sampling technique during the study period were considered as the study population. All mothers who gave birth in Gurage zone hospitals were included. Mothers who had critically ill, develop a childbirth complication, and unable to respond during data collection period were excluded. Single population proportion formula was used to calculate the final sample size. The following assumptions were considered; *p* = 30.9 %, the proportion of mothers who had given birth through caesarean section in North Wollo Zone public hospitals, Amhara region, Northeast Ethiopia [17], 95 % confidence interval (Zα/2 = 1.96), 4 % margin of error (d = 0.04), and 10 % non-respondent rate. Therefore, the final sample size was calculated to be 551. After reviewing two months of the obstetric case follow of all five hospitals, the final sample size was allocated to each hospital proportionally. Finally, the study subjects were selected using a systematic random sampling method based on the client flow in each hospital until reaching the final sample size.

### Study variables and measurements

The main outcome variable in this study was caesarean section delivery. Caesarean section (C-section) is defined as a comprehensive obstetric surgical incision in a women’s abdomen and uterus for delivery of the fetus, membrane, and placenta after viability of the fetus. Those mothers who had given birth through vaginal delivery includes instrumental deliveries (i.e. forceps and vacuum delivery) were coded as ‘1’ and those mothers who had given birth via CS coded as ‘0’ [17].

The explanatory variables for this study were includes; gravidity, categorized as ‘primigravida’, ‘multigravida’ and ‘grand multigravida’; parity, categorized as ‘nulliparous’ or ‘multipara’, ‘Grand multiparous’; fetal lie/presentation, categorized as ‘breech’ or ‘cephalic’, lie ‘transverse or oblique’; onset of labour, categorized as ‘spontaneous’, ‘induced or CS before labour’; malpresentation, categorized as ‘Yes’ and ‘No’; multiple pregnancies, categorized as ‘singleton’ or ‘multiple’; Gestational age, classified as ‘term > 37weeks’, ‘preterm < 37 weeks’ and ‘post-term > 42 weeks’ new-born birth weight; newborn birth weight, categorized as ‘normal birth weight (2500-4000gm)’, ‘low birth weight (< 2500gm)’, ‘macrosomia (> 4000gm)’; previous history of CS, categorized as ‘Yes’ and ‘No’[22, 23].

### Data collection tool and procedures

The tool was adapted after reviewing different relevant literatures [15–17, 24–29]. Firstly, the questionnaire was prepared in ‘English language’ and translated to ‘Amharic language’ by Amharic language experts and then translated backs to the ‘English language’ to check its consistency. The tool contains three parts; part 1, socio-demographic characteristics; part 2, obstetric related variables; and part 3, indications of performing caesarean section. Data were collected through reviewing of medical records of mothers and a structured, face-to-face exit interview was used. Five midwives (BSc) and one MSc health professionals participated in the data collections and supervision respectively. All relevant information including socio-demographic characteristics, obstetric related variables, and indications of performing caesarean section were collected.

### Data quality assurance

To ensure data quality, two days of training (one day for theoretical and one day for practical) were given for both data collectors and supervisor by principal investigator on the technique of the data collection process. A pre-test was done on 5 % of the sample size before data collection was processed and some modifications were done. The principal investigator and supervisor were closely follow-up the data collection process.

### Data processing and analysis

Data was clean, coded, and entered into Epi-data version 4.2.0 and exported into SPSS version 23 for further analysis. The magnitude of caesarean section was analysed. Data were presented in the form of frequency, mean, standard deviation, tables, pie-chart, and figures. Bivariate and multivariate analysis was done using the binary logistic regression model to identify factors associated with caesarean section deliveries. All statistically significant variables in bivariate logistic regression model were taken into account for multicollinearity tests. Multi-collinearity test was carried out using Variance Inflation Factor (VIF). Thus, the result of multi-collinearity test showed that there is no evidence of collinearity among the independent variables, since all included variables had VIF < 10. Hosmer-Lemeshow goodness tests were carried out to check model fitness (0.85). *P*-value < 0.05 results along with adjusted odd ratio 95 % confidence interval was used to declare a result as a statistically significant association.

## Results

### Socio-demographic characteristics

A total of 551 women were participated in this study and making a response rate of 98 %. The mean age of the respondents was 27.33 (± 4.83) years. Among a total study participants, 336 (61 %) were found in the age group between 25 and 34 years. Regarding maternal educational status, 211 (38 %) of mothers have completed primary education. Concerning marital status, 486 (88 %) women were married. Regarding residence, 400 (73 %) of study participants were lived in rural areas and 402 (73 %) of respondents were Gurage ethnicity. Concerning religion, 349 (63 %) of study participants were orthodox religious followers (Table 1).

**Table 1:** Socio-demographic characteristics of women’s who gave birth in Southwest Ethiopia, 2020

Variable	Frequency	Percentage
Maternal age		
15–24	152	28
25–34	336	61
35–44	63	11
Marital status		
Single	21	4
Married	486	88
Divorced/widowed	44	8
Maternal educational status		
No formal education	192	35
Primary education	211	38
Secondary education and above	148	27
Residence		
Urban	151	27
Rural	400	73
Maternal occupation		
Housewife	357	65
Merchant	77	14
Government employee	103	19
Daily labourer	14	3
Religion		
Orthodox	349	63
Muslim	157	29
Protestant	45	8
Ethnicity		
Gurage	402	73
Amhara	115	21
Oromo	34	6

### Obstetric related characteristics

The finding of this study showed that, 334 (61 %) and 402 (73 %) of mothers were multigravida and multiparous respectively. Out of the total respondents, 366 (66 %) of mothers had antenatal care visits and 211 (38 %) of mothers were utilized antenatal care services more than four times. About 302 (55 %) of mothers had practiced an antenatal care group. About 432 (78 %) of mothers had normal cephalic presentation and 415 (75 %) of mothers had experienced spontaneous onset of labour. Regarding gestational age and neonatal birth weight, 413 (75 %) of mothers gave birth at term (37–42 week) and 410 (74 %) of mothers had normal birth weight newborn (2500-400gm). Concerning the previous history of caesarean section, 69 (13 %) of mothers had history of previous caesarean section (Table 2).

**Table 2:** Obstetric related characteristics of women’s who gave birth in Southwest Ethiopia, 2020

Variables	Frequency	Percentage /%/
Gravidity		
Primigravida	92	17
Multigravida	334	61
Grand multigravida	125	23
Parity		
Primiparaous	82	15
Multiparaous	402	73
Grand multiparous	67	12
ANC visit		
Yes	366	66
No	185	34
Number of ANC visits		
1	55	10
2	44	8
3	56	10
≥ 4	211	38
Participation in antenatal care group		
Yes	302	55
No	249	45
Fetal presentation /lie		
Breech	72	13
Cephalic	432	78
Transverse/oblique	47	9
Onset of labour		
Spontaneous	415	75
Induced	78	14
No labour (pre-labour CS)	58	11
Multiple pregnancy		
Yes	96	17
No	455	83
Gestational age		
Term (37–42 week)	413	75
Preterm (< 37 week)	71	13
Post term (> 42 week)	67	12
Malpresentation		
Yes	129	23
No	422	77
Newborn birth weight		
Normal birth weight (2500-4000gm)	410	74
Low birth weight (< 2500gm)	78	14
Macrosomia (> 4000gm)	63	12
Previous history of caesarean section		
Yes	69	13
No	482	87

### Indication for performing caesarean section

**Fig. 1:**
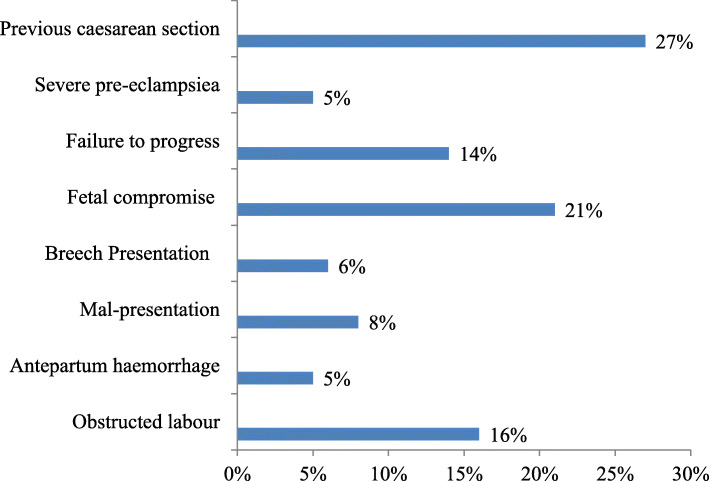
Absolute and relative indication of performing caesarean section among women’s who gave birth in Southwest Ethiopia, 2020

This study found that previous history of caesarean section 48 (27 %), and obstructed labour 28 (16 %) were the relative and absolute indications of performing CS respectively (Fig. 1).

### The magnitude of caesarean section

**Fig. 2:**
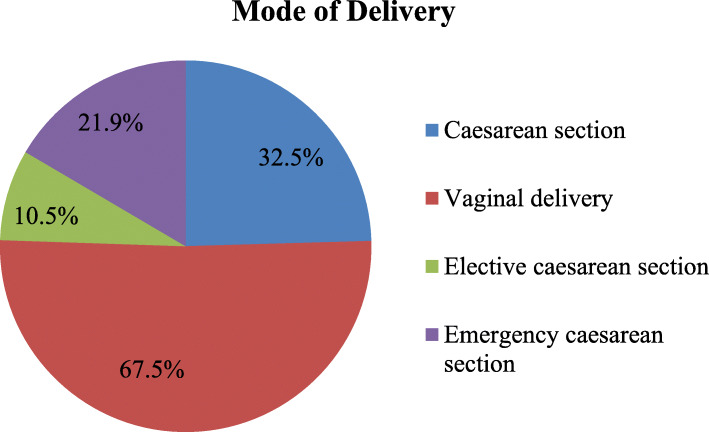
Magnitude of caesarean section delivery among women who gave birth in in Southwest Ethiopia, 2020

Out of the magnitude of caesarean section surgery 32.5 %, 10.5 %, and 21.9 % of mothers were given birth through elective and emergency caesarean sections respectively, while the magnitude of vaginal delivery was found to be 67.5 % (Fig. 2).

### Factors associated with caesarean section deliveries

The bivariate and multivariate logistic regression analysis model was done to identify factors associated with caesarean section delivery. In binary logistic regression model mothers resided in an urban area, multiple pregnancies, newborn low birth weight (< 2,5000gm), malpresentation, and previous history of caesarean section were significantly associated with caesarean section delivery.

In the multivariate binary logistic regression model analysis after controlling the confounders; mothers resided in an urban area, multiple pregnancies, malpresentation, and previous history of caesarean section were significantly associated with caesarean section delivery. Postpartum mothers who lived in urban areas were 2.58 times more likely to give birth through caesarean section delivery than those mothers who had lived in rural areas [AOR = 2.58, (95 % CI:1.66–4.02)]. Postpartum mothers who had been diagnosed with multiple pregnancies were 2 times more likely to give birth through caesarean section compared to those mothers who had single tone pregnancies [AOR = 2.15, (95 % CI:1.89–5.23)]. Postpartum mothers who had been diagnosed with malpresentation were 3 times more likely to give birth through caesarean section than their counterparts [AOR = 3.05, (95 % CI: 1.77–5.24)]. Postpartum mothers who had previous history of caesarean section were 2.5 times more likely to give birth through caesarean section than those women who did not have a history of previous CS [AOR = 2.55, (95 % CI:2.23–5.64)] (Table 3).

**Table 3:** Bivariate and multivariate logistic regression to identify determinate factors of caesarean section delivery among women’s who gave birth in Southwest Ethiopia, 2020

Variables	Caesarean Section Delivery		COR (95% CI)	AOR (95% CI)
	No (%)	Yes (%)		
Maternal age				
15-24	102(19)	50(9)	0.82(0.43-1.55)	1.15(0.54-2.46)
25-34	225(41)	111(20)	0.81(0.45-1.47)	1.03(0.52-2.05)
35-44	45(8)	18(3)	1	1
Maternal educational status				
No formal education	135(25)	57(10)	1	1
Primary education	140(25)	71(13)	0.8(0.79-1.97)	1.1(0.62-1.91)
Secondary and above	97(18)	51(9)	0.8(0.67-1.62)	0.9(0.53-1.54)
Place of residence				
Rural	298(54)	102(19)	1	1
Urban	74(13)	77(14)	3.04(2.06-4.49)*	2.58(1.66-4.02)*
Gravidity				
Primigravida	59(11)	33(6)	0.72(0.41-1.29)	0.717(0.37-1.38)
Multigravida	224(41)	110(20)	0.82(0.53-1.29)	0.81(0.48-1.35)
Grand multigravida	89((16)	36(7)	1	1
Parity				
Primiparaous	28(5)	54(10)	0.37(0.41-1.29)	0.72(0.37-1.38)
Multiparous	305(55)	97(18)	2.26(0.53-2.91)	2.81(0.68-3.35)
Grand multiparous	39(7)	28(5)	1	1
ANC visit				
Yes	239(43)	127(23)	0.74(0.5-1.08)	0.69(0.44-1.08)
No	133(24)	52(9)	1	
Multiple pregnancy				
Yes	71(13)	25(5)	1.45(1.27-5.64)*	2.15(1.89-5.23)*
No	301(55)	154(28)	1	1
Gestational age				
Term(37-42 week)	287(52)	126(23)	1	1
Preterm(<37 week)	45(8)	26(5)	0.76(0.78-1.23)	0.89(0.38-2.09)
Post term(>42 week)	40(7)	27(5)	0.65(0.431-1.701)	1.087(0.57-2.08)
Malpresentation				
Yes	101(18)	28(5)	2.01(1.96-4.99)*	3.05(1.77-5.24)*
No	271(49)	151(27)	1	1
Newborn birth weight				
Normal birth weight	278(51)	132(24)	1	1
Low birth weight	56(10)	22(4)	1.21(1.01-1.41)*	0.98(0.54-1.77)
Macrosomia	38(7)	25(5)	0.72(0.29-1.21)	0.55(0.25-1.21)
Pervious history of CS				
Yes	48(9)	21(4)	1.11(1.001-1.9)*	2.55(2.23-5.64)*
No	324(59)	158(29)	1	1

^*^p value<0.05, 1=ref., Hosmer-Lemeshow goodness tests=0.85

## Discussion

Caesarean section is a life-saving comprehensive obstetric procedure for women and newborn performed during pregnancy and childbirth-related complications [6]. However, inappropriate use of caesarean section can negatively affect women health [5]. This study found that a significant proportion of mothers were giving birth through caesarean section. The multivariate analysis shows that mothers resided in urban areas, multiple pregnancies, malpresentation, and previous history of caesarean section were significantly associated with caesarean section deliveries.

The present study revealed that the magnitude of CS was found to be 32.5 % indicated that almost twice more than the WHO recommendation of 10–15 % [6]. The finding was slightly higher than studies conducted in Felegehiwot referral hospital, Amhara region, Northwest Ethiopia 25.4 % [16], Nepal 22.6% [23], Vietnam 26.2 %, [26], Sri Lanka, India 25 % [11], Mizan-Aman general hospital, Southwest Ethiopia 21 % [25] and Shire, Tigray region, Northern Ethiopia 20.2 % [15]. The result was nearly consistent with studies conducted in North Wollo Zone public hospitals, Amhara region, Northeast Ethiopia 30.9 % [17], Hawassa university referral hospital, southern Ethiopia 32.8 % [18], and Jugal hospital, Harari Regional State, eastern Ethiopia 29.7 % [28]. However, it was lower than studies conducted in Addis Ababa public hospitals 38.3 % [14], Tanzania 49 % [10], and South Africa 42.4 % [8]. The possible explanation for the difference might be due to variation in socio-demographic characteristics of women, sample size determination, geographical location, and access to comprehensive obstetric health care services.

Postpartum mothers who had lived in urban areas were 2.58 times more likely to give birth through caesarean section than those mothers who had lived in rural areas. The result was consistent with a study conducted in North Wollo Zone public hospitals, Amhara region, Northeast Ethiopia [17], and Vietnam [26]. The reason might be those mothers who had lived in urban areas have better access to comprehensive obstetric health care service and they do have awareness of the long duration of labour pain. Therefore, due to fear of labour pain pregnant mothers might prefer to give birth through caesarean section compared to spontaneous vaginal delivery. However, the finding was inconsistent with a study conducted in Felegehiwot Referral Hospital, Amhara region, Northwest Ethiopia [16]. In Felegehiwot referral hospital those mothers who had resided in rural areas were more likely to give birth through caesarean section than those who had resided in urban areas. The possible explanation might be due to the difference in ANC service utilization. For instance, those mothers who lived in rural areas might have delayed initiation of ANC service, low healthcare seeking behaviours, and delayed decision making process, which might increase the risk of obstetric complication and in turn, increase the probability of giving birth through caesarean section.

Postpartum mothers who had been diagnosed with multiple pregnancies were nearly 2 times more likely to give birth through caesarean section compared to those mothers who had diagnosed with single-tone pregnancies. The possible explanation might be multiple pregnancies were associated with obstetric complications such as preterm labour, the premature rupture of membranes, malposition, and malpresentation of the fetus might increase the probability of giving birth through caesarean section. Additionally, health care workers might decide to perform caesarean section to avoid the risk of maternal and neonatal mortality associated with multiple pregnancies.

Postpartum mothers who had been diagnosed with malpresentation were 3 times more likely to give birth through caesarean section compared to their counterparts. The finding was in line with other studies conducted in Felegehiwot referral hospital, Amhara region, Northwest Ethiopia and North Wollo Zone public hospitals, Amhara region, Northeast Ethiopia [16, 17]. The reason could be due to the fact that malpresentation might be associated with prolonged labour, fetal distress, cephalopelvic disproportion, which might affects the progress of normal labour and in turn, physicians might prefer to perform caesarean section to increase the chance of maternal and neonatal survival.

Postpartum mothers who had a previous history of caesarean section were 2.5 times more likely to give birth through caesarean section compared to those mothers who did not have a previous history of caesarean section. The finding was comparable with a study conducted in North Wollo Zone public hospitals, Amhara region, Northeast Ethiopia [17], and Addis Ababa public hospitals [14]. The possible justification might be due to those women who had a history of previous caesarean section might develop antepartum haemorrhage, might have bad obstetric history, medical and surgical problems that compromise the effort or attempt of a trial of vaginal birth after caesarean section.

Our study also assessed the main indications of performing caesarean section. Thus, having previous history of caesarean section, fetal compromise, obstructed labour, and failure of labour progress was the leading indication of performing caesarean section. The finding was comparable with other studies conducted in Hawassa referral hospital, Eastern Ethiopia, Nepal, and South Africa [8, 18, 24]. The possible justification might be the fact that most of the leading indications of performing caesarean section listed in the above were medically acceptable reasons. For instance, those mothers who had previous history of caesarean section is a relative indication for caesarean section due to the risk of uterine ruptures. Fetal compromise, obstructed labour, and failure of labour progress were also an indication for caesarean section, because continuing spontaneous vaginal delivery might increase the risk of stillbirth and maternal mortality.

In general, maternal and neonatal mortality can be averted using medically justifiable caesarean section procedure. The predominant factors associated with caesarean section deliveries were identified such as mother resided in an urban area, multiple pregnancies, malpresentation, and previous history of caesarean section. This study has several strengths such as identifying leading indications and factors associated with caesarean section delivery in the study area and it used prospective data that in turn minimizes missing/incomplete data. However, this study was not void of limitations; for instance, lack of including health care provider perspective regarding the rising rate of caesarean section deliveries and it also shares limitation of cross-sectional study design.

## Conclusion

The overall magnitude of caesarean section was found high in the study area. Mothers resided in an urban area; multiple pregnancies, malpresentation, and previous history of caesarean section were significantly associated with caesarean section deliveries. Therefore, counselling of mothers on the risk of giving birth through caesarean section without absolute and relative medical indications and giving enough time particularly for those women who underwent a trial of vaginal birth after caesarean section is recommended.

## Data Availability

The data set used for this research is not available online. However, it is available upon reasonable request from the corresponding author.

## Abbreviations

ANC: Antenatal care
CS: Caesarean Section
APH: Antepartum Haemorrhage
SPSS: Statistical Package for Social Sciences
WHO: World Health Organizations
